# Cones Support Alignment to an Inconsistent World by Suppressing Mouse Circadian Responses to the Blue Colors Associated with Twilight

**DOI:** 10.1016/j.cub.2019.10.028

**Published:** 2019-12-16

**Authors:** Joshua W. Mouland, Franck Martial, Alex Watson, Robert J. Lucas, Timothy M. Brown

**Affiliations:** 1 Centre for Biological Timing, Faculty of Biology, Medicine & Health, University of Manchester, Oxford Road, Manchester M13 9PT, UK

**Keywords:** suprachiasmatic, photoreceptor, photoentrainment, color opponency, non-image-forming, metamer, daylight

## Abstract

In humans, short-wavelength light evokes larger circadian responses than longer wavelengths [1, 2, 3]. This reflects the fact that melanopsin, a key contributor to circadian assessments of light intensity, most efficiently captures photons around 480 nm [4, 5, 6, 7, 8] and gives rise to the popular view that “blue” light exerts the strongest effects on the clock. However, in the natural world, there is often no direct correlation between perceived color (as reported by the cone-based visual system) and melanopsin excitation. Accordingly, although the mammalian clock does receive cone-based chromatic signals [9], the influence of color on circadian responses to light remains unclear. Here, we define the nature and functional significance of chromatic influences on the mouse circadian system. Using polychromatic lighting and mice with altered cone spectral sensitivity (*Opn1mw*^*R*^), we generate conditions that differ in color (i.e., ratio of L- to S-cone opsin activation) while providing identical melanopsin and rod activation. When biased toward S-opsin activation (appearing “blue”), these stimuli reliably produce weaker circadian behavioral responses than those favoring L-opsin (“yellow”). This influence of color (which is absent in animals lacking cone phototransduction; *Cnga3*^*−/−*^) aligns with natural changes in spectral composition over twilight, where decreasing solar angle is accompanied by a strong blue shift [9, 10, 11]. Accordingly, we find that naturalistic color changes support circadian alignment when environmental conditions render diurnal variations in light intensity weak/ambiguous sources of timing information. Our data thus establish how color contributes to circadian entrainment in mammals and provide important new insight to inform the design of lighting environments that benefit health.

## Highlights

• Cone-derived blue:yellow color signals influence circadian entrainment in mice
• The blue colors associated with twilight suppress circadian light responses
• Color signals support circadian entrainment to low-amplitude light:dark cycles
• Color signals buffer the clock against cloud-related changes in light levels

## Teaser

Changes in the spectral content of ambient light are detectable to most mammals as a blue shift in the color of twilight. Mouland et al. show that these “blue” colors suppress circadian responses to light, supporting robust circadian entrainment when environmental conditions render light intensity a weak or unreliable indicator of time of day.

## Results

### Color Modulates Circadian Assessment of Light Levels

**Figure 1:**
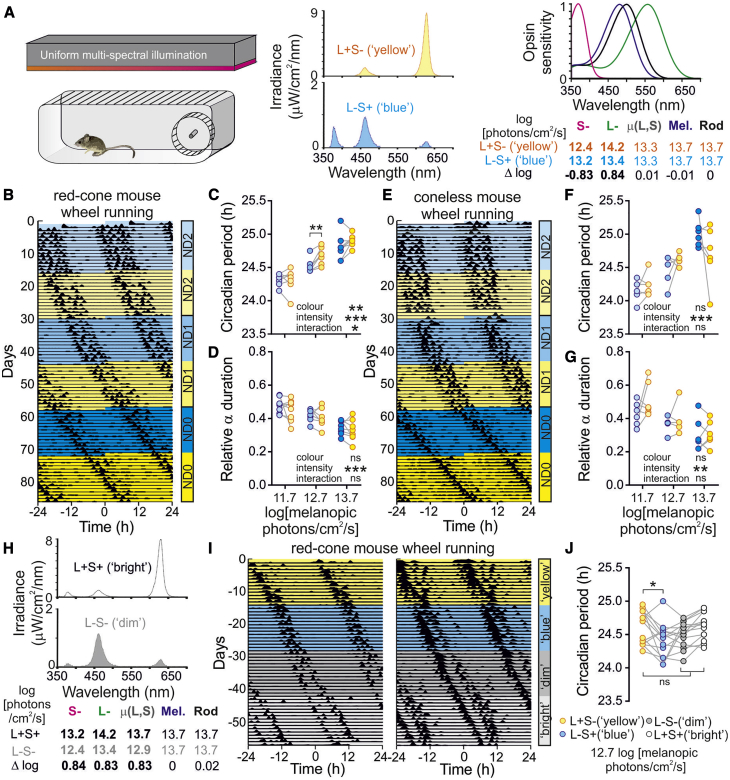
Color Modulates Circadian Assessment of Light Levels (A) Schematic of experimental paradigm (left), spectral composition of L−S+(blue) and L+S−(yellow) stimuli (mid), and opsin sensitivity curves (right) for red-cone mice with corresponding quantification for stimuli at maximum intensity (ND0). See also Figure S1 for additional details of stimulus design. (B) Representative actogram for red-cone mouse under constant L−S+(blue) or L+S−(yellow) illumination at 0.01, 0.1, and 1× intensity level shown in (A) (ND2–ND0, respectively). (C) Circadian period for red-cone mice under L−S+(blue) versus L+S−(yellow) illumination at varying intensity (n = 7–8/intensity). Data analyzed by two-way repeated measures (RM) ANOVA with Sidak’s post-tests: intensity, F_2, 20_ = 39.4; p < 0.001; color, F_1, 20_ = 11.7; p = 0.003; intensity × color, F_2, 20_ = 3.8; p = 0.04. (D) Activity bout duration (α), expressed as a fraction of circadian period length, for red-cone mice as above. Two-way RM ANOVA: intensity, F_2, 20_ = 17.0; p < 0.001; color, F_1, 20_ = 2.98; p = 0.10; intensity × color, F_2, 20_ = 0.1; p = 0.92. (E) Same as (B) but for coneless mouse. (F) Same as (C) but for coneless mice. Two-way RM ANOVA: intensity, F_2, 14_ = 31.3; p < 0.001; color, F_1, 14_ = 0.1; p = 0.82; intensity × color, F_2, 14_ = 2.6; p = 0.11. (G) Same as (D) for coneless mice (n = 5–6) mice. Two-way RM ANOVA: intensity, F_2, 14_ = 9.1; p = 0.003; color, F_1, 14_ = 2.4; p = 0.14; intensity × color, F_2, 14_ = 0.9; p = 0.44. (H) Spectral composition of stimuli that modulated cone illuminance (μ(L,S)) without changing color or melanopsin/rod excitation. (I) Representative actograms for two red-cone mice exposed to constant L+S−(yellow), L−S+(blue), L−S−(dim), and L+S+(bright) stimuli at ND1. (J) Circadian period determined for red-cone mice (n = 14) under the conditions illustrated in (I). Data analyzed by one-way RM ANOVA with Dunnett’s post-tests: F_3,39_ = 3.869; p = 0.016. ^∗^p < 0.05, ^∗∗^p < 0.01, and ^∗∗∗^p < 0.001; ns = p > 0.05.

Cone-derived color signals reach the suprachiasmatic nuclei (SCN) and can influence clock phase [9], but it remains unclear which colors most effectively engage circadian responses and how such a mechanism contributes to entrainment under real-world conditions. Given the predictable shifts in ambient light spectra at dawn and dusk [4, 12], we hypothesized that light whose color resembled twilight (i.e., blue) would produce weaker circadian responses than light of equivalent intensity but whose color was associated with daytime (yellow to white). To test this, we assessed circadian behavior under polychromatic lighting whose spectral composition could be varied to adjust color independently of light intensity (Figure 1A).

The mammalian circadian system tracks light intensity via a combination of melanopsin and outer-retinal signals relayed by intrinsically photosensitive retinal ganglion cells (ipRGCs) [4, 13, 14, 15]. Using the principles of silent substitution [16], we therefore aimed to generate stimuli with equivalent brightness for melanopsin, rods, and cones (termed here “equi-luminant”) but distinct spectra (and consequently color) for the dichromatic mouse visual system. To enable the generation of substantial differences in color while controlling melanopsin and rod activation, we employed a validated [9, 17, 18, 19, 20, 21, 22, 23, 24] mouse line (Opn1mw^R^; hereafter termed red-cone) [25], where the native M-cone opsin (λmax = 511 nm) is replaced with the human L-cone opsin (λmax = 556 nm).

We started by establishing a housing environment that provided diffuse overhead illumination from independently controllable light-emitting diode (LED) sources (Figure 1A). We then calibrated a polychromatic lighting condition (using 385-, 460-, and 630-nm primaries) that recreated a wild-type mouse’s experience of natural daylight (i.e., “white” light; Figure S1). By adjusting the intensities of each primary relative to this reference white point, we then produced a pair of experimental stimuli. The first maximized L-opsin and minimized S-opsin excitation (L+S−; therefore appearing “yellow” by analogy with the human long versus short-wavelength color channel). The second minimized L-opsin and maximized S-opsin activation (L−S+; therefore appearing “blue”) to recapitulate a wild-type mouse’s experience of twilight. Importantly, there were negligible differences (<0.01 log units) between L−S+ and L+S− stimuli in melanopsin and rod excitation as well as in the average illuminance for mouse cone opsins (Figure 1A).

We first used this approach to evaluate the impact of color on the circadian period of voluntary wheel running under constant illumination, a paradigm used extensively to assess the impact of light on the mammalian clock (e.g., [13]). Here, red-cone mice (n = 8) were exposed to alternating 2-week blocks of constant L−S+(blue) and then equi-luminant L+S−(yellow) illumination across 3 logarithmically spaced intensities (Figure 1B). As expected, circadian period reliably lengthened with increasing intensity, but we also identified a significant impact of color, with longer circadian periods under L+S−(yellow) versus L−S+(blue) illumination (Figure 1C), especially at intermediate intensities (Sidak’s post-test; p = 0.006). These data strongly support our hypothesis that blue light will have a weaker effect on the clock than equi-luminant yellow illumination.

Interestingly, another common impact of increasing light intensity on mouse behavior, compression of activity bout duration (α), was not similarly impacted. Hence, although there was a robust decrease in α as a function of intensity, we did not detect any significant influence of color (Figure 1D). This may reflect SCN-independent influences on activity [26] or the involvement of SCN neurons that process achromatic signals [9]. In either case, it seems that color does not globally impact all behavioral responses to light but instead more specifically impacts on clock speed.

Because our experimental stimuli selectively modulate the ratio of L- to S-cone opsin activation, circadian behavior should be indistinguishable under equi-luminant L+S− and L−S+ conditions in animals that lacked cone phototransduction (Figure 1E; *Cnga3*^*−/−*^ mice [27]; hereafter termed coneless). Accordingly, although coneless mice (n = 7) retained intensity-dependent increases in circadian period and reduction in α duration, there were no detectable effects of color (Figures 1F and 1G). Indeed, at the two highest intensities, coneless mice were at least as likely to display longer free-running periods under blue rather than equi-luminant yellow (7 out of 11 paired measurements), whereas this occurred in only 1 of 15 observations from red-cone mice (p = 0.003; Fisher’s exact test). By contrast, red-cone and coneless data were qualitatively similar at the lowest intensity (which falls below the range where strong cone-mediated responses are observable) [20].

We next sought to confirm that the reduction in the circadian period of red-cone mice under blue illumination at higher intensities was a specific result of color rather than a difference in effective cone illuminance. To this end, in a separate batch of red-cone mice (n = 14), we first presented 2-week blocks of L+S−(yellow) and then L−S+(blue) stimuli followed by blocks of two additional stimuli of intermediate color (equivalent to a wild-type mouse’s experience of an overcast day) but varying cone illuminance (Figure 1H; L+S+(“bright”) and L−S−(“dim”)). Effective photon flux for melanopsin and rods was 12.7 log photons/cm^2^/s for all stimuli. Our expectation was that, if the reduced circadian period under L−S+(blue) illumination simply reflected a reduction in effective cone illuminance, circadian periods should be even more reduced under the L−S− (dim) condition. As above, we once again found a significant decrease in circadian period under L−S+(blue) versus L+S−(yellow) illumination (Figures 1I and 1J; Dunnett’s post-test; p = 0.04). By contrast, circadian periods were not significantly different from L+S− under either L−S−(dim) or L+S+(bright) conditions (p = 0.44 and p = 0.91, respectively). Collectively, these data confirm a specific impact of cone-derived chromatic signals on circadian period, with colors resembling those encountered during late stages of twilight (blue) exerting a weaker impact on the clock than colors associated with daytime illumination.

### Color Modulates Re-entrainment following “Jet Lag”

Our data above indicate that the twilight blue shift substantially attenuates circadian responses to light and thus imply that blue stimuli should be less effective at resetting the clock than equi-luminant yellow. To test this, we initially evaluated changes in the timing of red-cone mouse (n = 16) behavioral rhythms in response to acute pulses of L+S−(yellow) versus L−S+(blue), presented immediately following transfer from a light:dark (LD) cycle to constant dark. We chose this approach to avoid long-term adaptation effects that might accompany testing under constant dark housing (where resetting responses are dominated by rod contributions) [13]. With the aim of further increasing cone influences, we employed brief (5-min) exposures [13, 28] at sub-saturating intensities and presented these either early or late in the projected night (Figures S2A and S2B). Despite a trend toward smaller phase advances and delays following blue stimuli, in neither case were the measured shifts significantly different from those evoked by yellow (Figure S2C). Given the inter-trial variability associated with this kind of assay [29, 30, 31], it is hard to definitively exclude any impact of color. Nonetheless, it seems that, under the specific conditions studied here, color does not exert a major influence on the magnitude of acute light-pulse-induced resetting.

**Figure 2:**
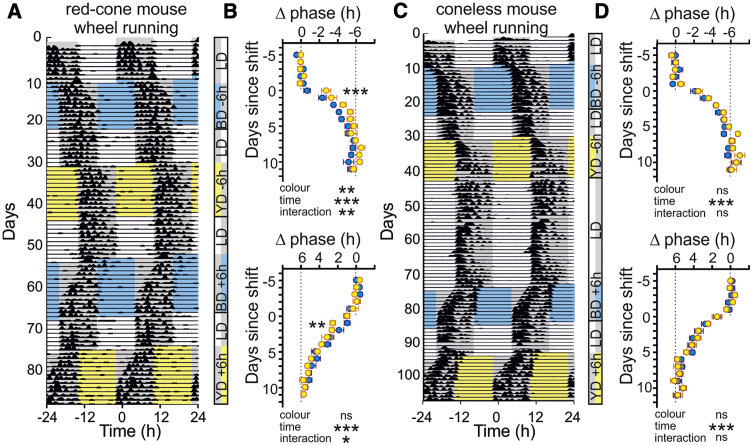
Color Modulates Re-entrainment following Jet Lag (A) Representative actogram for red-cone mouse under 12:12LD cycles and subsequently exposed to 6-h delays and advances where the light phase was rendered in L−S+(blue) or L+S−(yellow) at 0.1× intensity levels shown in Figure 1A. (B) Mean ± SEM phase change (mid-point between activity onsets and offsets, normalized to pre-shift average for each mouse) for red-cone mice (n = 8) during L−S+(blue) and L+S−(yellow) shifts. Data analyzed by two-way RM ANOVA with Sidak’s post-tests are shown. Delays (top panel): time, F_16,112_ = 103.5; p < 0.0001; color, F_1, 7_ = 24.2; p = 0.002; color × time, F_16, 112_ = 2.3, p = 0.007. Advances (bottom panel): time, F_16,112_ = 99.3; p < 0.0001; color, F_1, 7_ = 2.45; p = 0.16; color × time, F_16, 112_ = 1.7; p = 0.049. (C) Same as (A) but for coneless mouse. (D) Same as (B) but for coneless mice. Two-way RM ANOVA is shown. Delays (top panel; n = 7): time, F_16, 96_ = 143.8; p < 0.0001; color, F_1, 6_ = 5.17; p = 0.06; color × time, F_16, 96_ = 1.60; p = 0.08. Advances (bottom panel; n = 8): time, F_16, 112_ = 133.2; p < 0.0001; color, F_1, 7_ = 0.05; p = 0.84; color × time, F_16, 112_ = 0.56; p = 0.91. ^∗^p < 0.05, ^∗∗^p < 0.01, and ^∗∗∗^p < 0.001, respectively; ns = p > 0.05. See also Figure S2 for details of responses to acute pulses of L−S+(blue) and L+S−(yellow) stimuli.

Because we identified clear effects of color under much longer durations of illumination than those used above (Figure 1), we next asked whether color would modulate the ability of mice to re-entrain to large shifts in the timing of the LD cycle (jet lag paradigm). Here, red-cone mice (n = 8) experienced at least 7 days of a conventional 12:12 LD cycle and then the onset of the light phase was delayed or advanced by 6 h and rendered as either L−S+(blue) or L−S+(yellow) (Figure 2A). We found that changes in phase (activity midpoint) produced by L+S−(yellow) stimuli were significantly more rapid than L−S+(blue) for both delay and advance shifts (Figure 2B). By contrast, we could not detect a significant influence of color for either shift direction in coneless mice (Figures 2C and 2D). Collectively, these data support our hypothesis that color signals, supplied by cones, modulate circadian responses to light such that stimuli that appear blue are less effective at re-entraining the circadian system than those with yellow color.

### Color Supports Circadian Entrainment to Unreliable Intensity Cues

Our data provide a straightforward mechanism by which color signals could aid circadian entrainment—by reducing responses to light whose color is indicative of late stages of twilight. To probe the ecological significance of this mechanism, we next established a new housing environment that allowed more dynamic control over the intensity and color of illumination and fitted this with passive infrared sensors that detect even small wake-related behaviors rather than just daily variations in locomotion [32].

Using this system, we first asked whether a primary function of color input was to support circadian entrainment when diurnal changes in light intensity are small. In the natural world, especially in the regions where the ancestors of laboratory mice evolved, this is an uncommon circumstance. Nonetheless, such a possibility has been proposed to explain how some animals maintain entrainment during the arctic summer, where daily variations in light intensity are very markedly reduced [12]. Moreover, given the reduced exposure to natural light associated with modern life, such an effect of color (if present) could have substantial practical significance.

**Figure 3:**
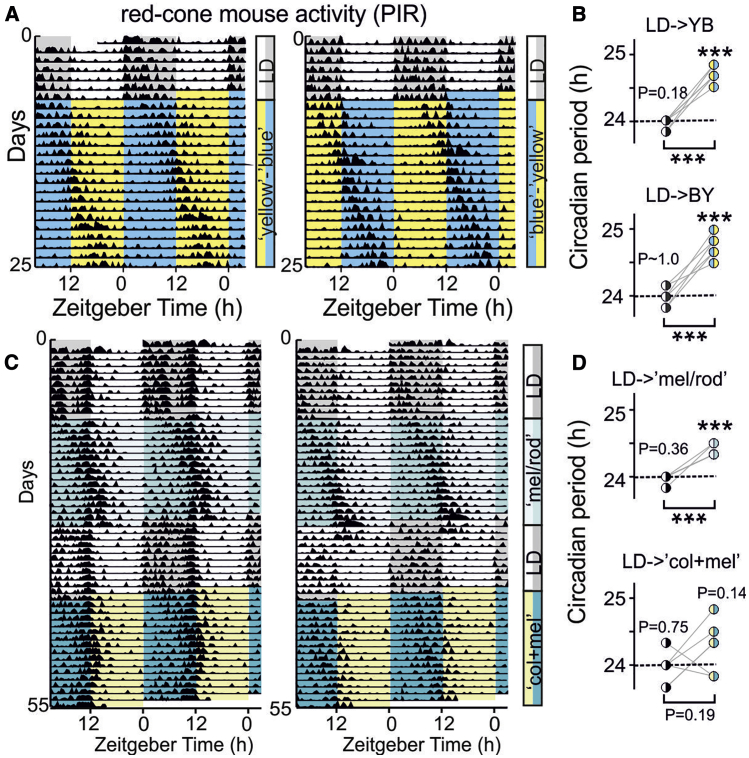
Color Is Not an Independent Timing Cue for the Circadian Clock (A) Representative passive infrared (PIR)-derived actograms for two red-cone mice transferred from 12:12LD to aligned L+S−:L−S+ (yellow:blue) or L−S+:L+S− (blue:yellow) cycles (spectra provided in Figure S3A). (B) Period of activity rhythms under LD and L+S−:L−S+ (yellow:blue; top) or L−S+:L+S− (blue:yellow; bottom). Data (n = 6 in both cases) are compared against an expected period of 24 h (one-sample t tests) and between conditions (paired t tests), showing an increase in period, above 24 h, in both cases. (C) PIR-derived actograms for two red-cone mice transferred from 12:12LD to aligned cycles providing modest daily changes in illumination just for melanopsin and rods (mel/rod; Figure S3B) or with superimposed changes in color (col+mel; Figure S3C). Note, two mice retained partial entrainment under col+mel (shown in left panel and Figure S3D) although other animals free ran with a long circadian period (representative example in right panel). (D) Period of activity rhythms under LD and subsequent mel/rod (top) or col+mel cycles (bottom). Data (n = 6 in both cases) are analyzed with one-sample t tests and paired t tests as above. ^∗∗∗^p < 0.001. Spectral power distributions for all stimuli are provided in Figure S3.

In initial experiments, we evaluated whether mice could maintain entrainment in the presence of large diurnal variations in color without any associated change in light intensity. Accordingly, we first entrained mice to a conventional 12 h:12 h LD cycle and then replaced the light phase with either L+S−(yellow) or equi-luminant L−S+(blue) and the dark phase with the opposite color (Figure 3A; n = 6/condition; spectra in Figure S3A). In both cases, mice immediately lost entrainment and free ran with an elongated period (Figure 3B). Thus, even fairly large variations in color do not act as an independent zeitgeber for the circadian system, implying that color instead exerts its effects by modulating responses to variations in light intensity.

We next then investigated whether daily changes in color would facilitate entrainment to very low amplitude diurnal variations in light intensity by generating two new sets of lighting conditions. The first provided a modest (0.75 log unit) daily variation in intensity for melanopsin and rods (“mel/rod”; Figure S3B), with no change in color or cone illuminance. The second provided an equivalent daily variation in melanopsin/rod activation while presenting a simultaneous change in color (“col+mel”; blue color aligned with the dim phase; Figure S2C). As expected, on transition to the mel/rod condition, red-cone mice (n = 6) immediately lost entrainment (Figures 3C and S2D) and began to free run with a long period (Figure 3D). By contrast, this disruptive effect of a reduced diurnal variation in light intensity was ameliorated by inclusion of color changes. Specifically, although no animals showed un-interrupted entrainment following the switch form LD to col+mel, two animals retained ∼24-h rhythms indicative of partial entrainment (Figures 3C and S2D; remaining animals free ran; Figure 3C). As a result, across the group, we did not detect a significant period lengthening (Figure 3D), as in the other conditions tested here.

In summary, these data provide some support for the idea that color may aid entrainment to low-amplitude light dark cycles but suggest that, for robust entrainment, changes in light intensity greater than those achievable here are required. In fact, even during the arctic summer, the diurnal change in light intensity will be at least double that which we employed (Figure S3E), although much of the diurnal color change would also be lost (at least for mice). Accordingly, although we do not discount the idea that some animals use color to help entrainment under such conditions, it seems unlikely that this is the primary role of color input to the clock for most mammals. Instead, a more globally relevant potential benefit of using color is to compensate for stochastic fluctuations in the diurnal rhythm of light intensity, e.g., due to variations in cloud cover [11, 12].

**Figure 4:**
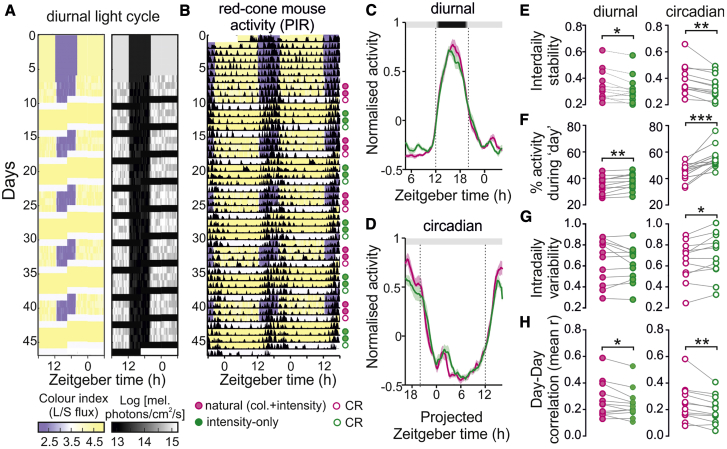
Daily Changes in Color Support Stable Entrainment in the Face of Weather-Related Variation in Light Intensity (A) Schematic of the light exposure paradigm that included naturalistic changes in color and intensity with superimposed stochastic variations to simulate clouds. Left and right panels, respectively, provide quantification of apparent color and concurrent changes in light intensity; see Figure S4 for additional details of stimuli. (B) Representative PIR-derived actograms for a red-cone mouse under the lighting schedule shown in (A). Symbols adjacent to the traces indicate 24-h epochs that were used for subsequent analysis. (C) Mean ± SEM normalized activity waveforms for red-cone mice (n = 12) under days providing natural changes in color and intensity or matched intensity-only days. (D) Same as (C) but for 24-h epochs of constant dim illumination following natural or intensity-only days. (E–H) Quantification of rhythm robustness and stability for red-cone mice (n = 12) under natural or intensity-only days (diurnal) and subsequent constant routine (circadian), analyzed throughout by paired t test; (E) interdaily stability (diurnal: p = 0.02; circadian p = 0.001); (F) percent activity occurring during the “day”/projected day (diurnal: p = 0.004; circadian p = 0.0002); (G) intradaily variability (diurnal: p = 0.26; circadian = 0.02); (H) mean day-day correlation in activity patterns (diurnal: 0.025; circadian: 0.005). See STAR Methods for further details of analysis procedures. ^∗^p < 0.05, ^∗∗^p < 0.01, and ^∗∗∗^p < 0.001.

Clouds can reduce ambient light levels by >10-fold, rendering the timing of sunrise/sunset ambiguous for a system that relies simply on light intensity. The twilight blue shift, however, is retained irrespective of clouds [9, 10, 11]. To test whether this color information buffers clock entrainment against weather-related changes in illumination, we designed an experimental paradigm to provide naturalistic cycles of color and/or light intensity that incorporated stochastic variations to simulate the impact of clouds (Figure 4A). Our lighting system allowed us to recreate (for red-cone mice) much of the natural variation in color and light intensity that a wild-type mouse would experience around dawn and dusk on clear and cloudy days (Figures S4A–S4D). We then presented such stimuli as cycles of 3 days, modeled on a northern latitude summer, with continuously varying changes in cloud cover (Figures S4D and S4F; “natural”). For comparison, we followed these with matched cycles providing identical daily changes in light intensity but where the color was fixed throughout to resemble day (Figures S4E and S4F; “intensity only”).

Red-cone mice (n = 12) were then initially entrained to a 16 h:8 h LD cycle (providing stable daily changes in color and light intensity) and thereafter experienced naturalistic daily variations in light intensity with simulated clouds that included or lacked the associated variations in color (Figures 4A and 4B). Analysis of the mean daily activity patterns revealed consistent changes under natural versus intensity-only cycles. Specifically, although the overall timing was similar under both conditions (Figures 4C and S4G), the magnitude of the diurnal variation in activity was compressed in the absence of color signals (Figure 4C). To quantify this effect, we used an established metric of circadian rhythm robustness (“interdaily stability”) [33], confirming a significant impairment under intensity-only versus natural days (Figure 4E).

This observation could reflect either a simple reduction in the magnitude of daily rhythms in the absence of color or an increase in the day-day variability of these activity patterns. Subsequent analyses implicated both factors. Hence, the percentage of daily activity that occurred outside the night was increased under intensity-only (Figure 4F), while bin-bin variation in activity (“intradaily variability”) [33] was equivalent under both conditions (Figure 4G). Thus, the intensity-only condition was associated with a reduction in the amplitude of day-night variations in activity without any substantive increase in the fragmentation of activity patterns across the daily cycle. Importantly, however, when we analyzed the day-day similarity of activity patterns (by calculating the mean pairwise correlations), we found a significant reduction under intensity-only days (Figure 4H), indicating that daily activity timing was more variable in the absence of color signals.

In summary, naturalistic diurnal variations in color confer enhanced robustness and stability to daily activity patterns in the face of weather-related fluctuations in light intensity. To determine whether this results from a specific impact on the circadian control of activity, interspersed between blocks of natural and intensity-only days, we also included 24-h epochs of constant dim illumination of intermediate color (Figures 4A and 4B). Importantly, in the absence of environmental cues, mean daily activity patterns exhibited a further compression in amplitude when animals had previously experienced intensity-only versus natural days (Figure 4D). Accordingly, subsequent quantification revealed a similar set of changes to those described above but of greater magnitude. Specifically, interdaily stability was significantly reduced following intensity-only versus natural days (Figure 4E), and this effect was associated with significant increases in the percentage of daily activity occurring outside the projected night (Figure 4F), increased intradaily variability (Figure 4G), and a reduction in mean day-day correlation (Figure 4H). These data thereby confirm that naturalistic variations in color substantially enhance the amplitude and stability of clock-driven behavioral rhythms when the diurnal variation in light intensity provides unreliable timing information.

## Discussion

Contrary to common beliefs, it is yellow rather than blue colors that have the strongest effect on the mammalian circadian system. This relationship aligns with natural shifts in the color of ambient illumination, detectable during twilight by mammals with di- and tri-chromatic visual systems [12]. Accordingly, we show that this color signal supports robust and stable circadian-driven behavior in the natural world, where stochastic variations in light levels introduce ambiguity to intensity as a signal of time of day.

Theoretically, reduced circadian responses to blue colors could arise indirectly as a result of chromatic changes in pupil diameter; however, our previous work indicates that chromatic blue:yellow modulations do not produce observable pupillary responses in mice [17]. By contrast, our identification of a significant proportion of cells within the SCN that process cone-derived chromatic signals [9] provides a simple and direct neurobiological origin for the effects of color reported here.

Although the observed reductions in circadian responses to stimuli that resemble twilight are logical from an ecological perspective, this effect is surprising given the overall positive relationship between SCN firing and circadian resetting [20, 34] and the fact that most chromatic SCN cells are excited by blue colors [9]. The implication then is that these “blue-ON” SCN cells, whose responses align with those of a recently identified subtype of chromatic ipRGC [35], may actively oppose circadian phase resetting. Because vasopressin-expressing SCN neurons are believed to oppose light-driven circadian resetting [36], an intriguing possibility is that this population corresponds to those that process blue-ON signals [4].

An especially pertinent question, however, is whether the effects of color described here extend to other mammals, such as humans. The qualitative relationship between sun position and blue-yellow color should be retained for any mammal capable of color vision [12], and theoretical studies suggest that color could aid circadian entrainment in humans [11]. Existing evidence for color opponency in primate ipRGCs and melanopsin-dependent responses in man [37, 38, 39, 40] give further reasons to believe that the effects of color reported here could extend also to humans. To date, however, much of our current understanding of the spectral sensitivity of the human circadian system has been inferred based on acute “non-visual” responses, such as melatonin suppression. Consistent with a very recent observation that S-cone selective modulations do not noticeably influence such responses [41], acute suppression of melatonin by light appears to be primarily driven by melanopsin [42]. Such responses do not always provide a reliable proxy for circadian photosensitivity [43], however. Indeed, direct investigations of human circadian resetting reveal that low-intensity, short-wavelength light (460 nm) produces smaller responses than longer wavelength light (555 nm) of equivalent melanopic illuminance [3]. These data are therefore consistent with the circadian effects of color we identify in mice.

Such an arrangement is potentially important for practical approaches intended to adjust the circadian impact of artificial light. Current approaches typically rely on manipulating the ratio of short- and long-wavelength light, achieving modest differences in melanopic illuminance at the expense of perceptible changes in color [44]. As a result, stimuli with high melanopsin excitation appear “bluer” (and vice versa). A strong prediction of our research is that these changes in color may oppose any benefits obtained from modulating melanopsin photon capture. Recent work indicates that melanopsin-directed modulations that lack perceptible difference in color exert beneficial effects [45, 46]. Our data now suggest that supplementing such approaches with color changes of the appropriate direction could be especially effective at modulating circadian responses.

## STAR★Methods

### Key Resources Table

**Table:** 

REAGENT or RESOURCE	SOURCE	IDENTIFIER
**Experimental Models: Organisms/Strains**		

Mouse: *Opn1mw*^*R*^	Dr Jeremy Nathans, Johns Hopkins University	MGI Cat# 2678771, RRID:MGI:2678771
Mouse: *Cnga3*^*−/−*^		MGI Cat# 3723602, RRID:MGI:3723602

**Software and Algorithms**		

The Chronobiology Kit: KitCollect, KitMonitor, KitAnalyzer	Stanford Software Systems	https://query.com/chronokit/
MATLAB R2017a	MathWorks	https://uk.mathworks.com/products/matlab.html
GraphPad Prism 7.04	GraphPad	http://www.graphpad.com/
Python v2.7.10	Python	https://www.python.org/downloads/

**Other**		

RGB LED strips	Expert Electrical Supplies	LEDST60RGB
UV LED Strips	Expert Electrical Supplies	LEDST60UV/385NM
RGBW bulbs	LIFX	LIFX Color 1000
UV bulbs	Led Engin	LZ1-00UA00-00U7
405nm LED	Thorlabs	M405L4
460nm LED	Thorlabs	M455L4
630nm LED	Thorlabs	M625L4
PTFE diffusing sheet	Direct Plastics	PTS01004
Neutral density gel 211 0.9ND	Lee Filters	http://www.leefilters.com/lighting/colour-details.html#211
Avian D Coating, White paint	Avian Technologies	https://aviantechnologies.com/product/avian-d-white-reflectance-coating/
COMPASS Passive infrared system (PIR)	Lawrence Brown [32]	NA

### Lead Contact and Materials Availability

This study did not generate new unique reagents. Further information and requests for resources should be directed to and will be fulfilled by the Lead Contact, Tim Brown (Timothy.Brown@manchester.ac.uk).

### Experimental Model and Subject Details

#### Animals

All experiments received institutional ethics committee approval and in accordance with UK Animals (Scientific Procedures) Act 1986, and European Directive 2010/63/EU. Adult (> 8 weeks) male mice from a C57BL/6 background strain were used throughout. For the majority of the experiments we used mice expressing the human L-cone opsin in place of their native M-cone opsin (*Opn1mw*^*R*^ [25];). Additional experiments used mice lacking the cone-specific cyclic nucleotide gated channel alpha subunit (*Cgna3*^*−/−*^) [27]).

Mice were individually housed with *ad libitum* food and water. Cages were located in light tight cabinets where the ambient light could be carefully controlled. Under several paradigms (Constant light, and both phase shifts experiments) mice were also housed with access to a running wheel to assess locomotor activity.

### Method Details

#### Light Sources

##### Housing environment 1

One light tight cabinet (used to generate data in Figures 1 and 2) was fitted with four parallel rows of RGB (SMD5050) and ultraviolet (385nm) LED strips LED intensities with drivers to provide pulse-width modulation based regulation of LED intensity (Expert Electrical Supplies Ltd.; Rochdale, UK). A 1.5mm thick PTFE diffusing sheet (Direct plastics; Sheffield, UK) was installed ∼5cm below the LEDs to provide uniform illumination. Neutral density (ND) gels sheets (Lee Filters; Andover, UK) were fitted to the diffuser to reduce light intensity by 10 or 100 fold as required (ND1 and ND2 respectively).

##### Housing environment 2

Four custom built light boxes were affixed above the roof of the second cabinet. Each light box consisted of two smart RGBW bulbs (LIFX A60; LIFX, Cremorne, Australia) and 6 violet bulbs (405nm, Led Engin LZ1-00UA00-00U7; RS Components, Manchester UK), to allow for 5-primary illumination. A PTFE diffuser was mounted to floor of light boxes/roof of the cabinet and the interior of the cabinet was painted white to provide uniform illumination. The LIFX bulbs were connected wirelessly over a local network, UV bulbs were connected to LED drivers (T-Cube; Thorlabs, Ely, UK) via a multichannel analog output module (NI 9264; National Instruments, TX, USA). LED intensities were then controlled on a second by second basis using a PC running Python (2.7.10). Neutral density gels were added to each box to adjust the overall brightness as required.

##### Phase re-setting chamber

Stimuli were presented via a custom light source (components from Thorlabs) consisting of three independently controllable LEDS (405nm, 460nm and 630nm) combined by dichroic mirrors. Light stimuli were then and projected (via a concave lens) onto a 1.5mm thick PTFE diffusing sheet (Direct plastics) that sat on top of a cylindrical chamber coated with spectrally neutral reflective paint (Avian D coating, Avian Technologies LLC, NH, USA).

#### Light Stimuli

Stimuli were designed and calibrated as described previously [9, 17] using calibrated spectroradiometers (DMc150; Bentham Instruments Ltd, UK, and SpectroCal, Cambridge Research Systems, UK). In brief, photon absorption for each photopigment was calculated using Govardovskii nomograms ([47] with peak absorbance at: S-opsin, 365nm; L-opsin: 556nm; melanopsin, 480nm; rhodopsin, 498nm) adjusted for lens transmission [48]. In most cases, stimuli were designed to provide identical excitation of melanopsin and rods and to provide the same average illuminance for L- and S-cone opsin but to differ in the ration of L- versus S-opsin excitation (i.e., color). In other cases, stimuli were designed to modulate illuminance for cones (or rod/melanopsin excitation) without changing color, or to simultaneously modulate color and intensity. Spectra and relevant quantification for all stimuli are provide in the relevant figures or associated supplemental figures.

#### Behavioral Paradigms

For experimental paradigms 1-3 outlined below (data shown in Figures 1 and 2 and associated supplemental figure), mice were housed with a running wheel to assess locomotor activity. Wheel revolutions were acquired in 60 s bins using The Chronobiology Kit (Stanford Software Systems, Santa Cruz, CA). Mice were housed with the running wheel for at least one week before starting the experimental protocols. In most cases, light exposure and data acquisition was performed in housing environment 1 with the exception of acute phase resetting assays (see below). For experimental paradigms 4-6, we used housing environment 2 and measured cage activity via a passive infrared (PIR) system, as described previously [32].

##### 1. Effect of color on clock speed

Eight *Opn1mw*^*R*^ mice aged 23 weeks and 7 *Cgna3*^*−/−*^ mice aged 14-19 weeks were put into constant light conditions at ND2. Every two weeks the lights alternated between L-S+(‘blue’) and L+S-(‘yellow’) conditions (see spectra and quantification in Figure 1A), and every 4 weeks an ND gel was removed from the lights increasing the irradiance by 10-fold. A second batch of mice (14 *Opn1mw*^*R*^ mice aged 10-15 weeks) were housed under constant light conditions (at ND1) and received 2 successive 2 week blocks of L+S-(‘yellow’), L-S+(‘blue’), L-S-(‘dim’) and L+S+(‘bright’) cone selective stimuli (see Figure 1H).

##### 2. Jet-lag paradigm

Eight *Opn1mw*^*R*^ mice aged 14-22 weeks and 8 *Cgna3*^*−/−*^ mice aged 9-17 weeks were housed under a conventional 12h:12h LD cycle (fluorescent lighting; effective photon flux = 14.5, 14.7, 15 and 13 log photons/cm^2^/s for melanopsin, rhodopsin, L-cone and S-cone opsin respectively). After one week the mice were then subjected to a 6 hour phase delay or phase advance with the light phase rendered in either L-S+(‘blue’) or L+S-(‘yellow’) as in Figure 1A. Following re-entrainment (> 12 days) to the new LD cycle, mice were returned to conventional LD for at least 1 week prior to the next stimulus.

##### 3. Acute phase re-setting

Sixteen *Opn1mw*^*R*^ mice aged 6-16 weeks were housed under standard 12h:12h LD for 2 weeks. Subsequently, during early (ZT14-15) or late night (ZT21-22) mice were transferred to a test chamber (described above) where they were illuminated for 5 minutes with L-S+(‘blue’) or L+S-(‘yellow’) illumination (spectra and quantification in Figure S2A). Transfer was performed in darkness using IR goggles (ATN NVG-7, Armasight Inc., NH, USA) and subsequently mice were returned to their home cage under constant darkness for a further 10 days. Mice were then returned to 12h:12h LD for two weeks prior to receiving another test stimulus.

##### 4. Color-only entrainment paradigm

*Opn1mw*^*R*^ mice (n = 6/condition) aged 16-19 weeks were housed for a week under a 12h:12h LD cycle (light phase effective photon flux = 13.9, 13.8, 14, 12.9 log photons/cm^2^/s for melanopsin, rhodopsin, L-cone and S-cone opsin respectively). After entraining to these conditions, the LD cycle was changed to a 12h:12h ‘yellow’:’blue’ or ‘blue’:’yellow’ cycle (spectra and quantification in Figure S3A).

##### 5. Color with low amplitude diurnal lighting changes

Six *Opn1mw*^*R*^ mice aged 10-11 weeks were housed for a week under a 12h:12h LD cycle (day component was the ‘mel/rod+’ stimuli from Figure S3B). Subsequently the dark phase of the LD cycle was replaced by a ‘mel/rod-‘ stimulus (Figure S3B) such that the diurnal light cycle provided a modest difference in melanopsin and rod illumination but no change in color or cone illuminance. After 2 weeks, mice were returned to LD to re-entrain for 10 days. Finally, mice were transferred to a new diurnal cycle that provided an identical change in melanopsin and rod excitation to that used above but which also incorporated large changes in color (spectra and quantification in Figure S3C).

##### 6. Natural Entrainment paradigm

Two batches of 6 *Opn1mw*^*R*^ mice (11-13 & 15-18 weeks) were used for this experiment. Mice were housed under a 16h:8h lighting cycle that provided a daily variation in color and intensity resembling changes occurring between solar elevations of +6 and −4 degrees relative to the horizon (Figures S4A and S4B). Mice subsequently experienced 5 repeating epochs consisting of i) a 3 day block of naturalistic color and intensity changes with smooth twilight transitions and continuously varying fluctuations simulating clouds or ii) a matched 3-day block providing identical changes in light intensity but where color was fixed to resemble day (See Figures 4A and S4D–S4F). Each block was terminated by a 24h epoch of constant dim illumination of intermediate color. For the natural and intensity only cycles described above fluctuations simulating clouds were randomly generated *a priori* such that individual days were distinct from any other day under the same condition.

### Quantification and Statistical Analysis

All statistical analyses were performed using (GraphPad Prism 7.04; GraphPad Software Inc., CA, USA), with criteria for significance set at p < 0.05. Sample size and the other relevant statistical details are provided in the main text.

#### 1. Effect of color on clock speed

Circadian period was determined by χ^2^-periodogram [49], performed on 2 week blocks of data under each experimental condition. Activity bout duration (α) was defined as the faction of each circadian day that activity was above the mean (by reference to period as determined above). For subsequent statistical comparison of effects of L-S+(‘blue’) versus L+S-(‘yellow’) stimuli at varying irradiance (Figures 1B–1G), data were then analyzed by 2-way RM ANOVA with color as a repeated factor and Sidak’s post-tests where ANOVA revealed significant main effects of color or interaction with intensity. Data analyzed for each intensity excluded those cases where an individual exhibited negligible or interrupted wheel running under one or both of the two tested colors (due to blocked wheels). This was the case for 1 red-cone mouse (ND1 data excluded) and two coneless mice (for one individual ND2 and ND1 data excluded, for the second ND0 and ND2 data excluded). Of the remaining coneless data, one animal exhibited an unexpectedly short free-running period under high intensity ‘yellow’ illumination. In the absence of any overt technical reason to reject this data, it is included in the analysis. Of note, however, re-analysis (2-way RM ANOVA) excluding ND0 data from that individual produced an outcome equivalent to that reported in the text: a significant effect of irradiance (F_2, 13_ = 36.2; p < 0.0001) but not color (F_1, 13_ = 0.84; p = 0.38) or interaction with irradiance (F_2, 13_ = 1.94; p = 0.18).

For comparison of circadian period effects of color and-cone illuminance signals (performed at ND1 only, Figures 1I and 1J) data was analyzed by one-way RM ANOVA with Dunnett’s post-tests (no data excluded).

#### 2. Jet-lag paradigm

Circadian phase was assessed as the midpoint between activity onset and offset on each day (respectively defined as the start of a 30min epoch where activity exceed the daily mean and the start of a 90min epoch where activity fell below the daily mean). For analysis, circadian phase markers for each mouse were normalized by subtracting the mean for the 5-day epoch preceding a shift in the LD cycle. The data for L-S+(‘blue’) and L+S-(‘yellow’) stimuli under each condition were then analyzed by two-way RM ANOVA, with Sidak’s post-tests where we detected main effects of color or color X time interactions. For analyses of red-cone mice there were no exclusions. For analyses of coneless mice, one animal was excluded from analysis of delay shifts due to poor running during the first LD epoch that prevented reliable determination of starting phase prior to ‘blue’ delay. Analysis performed as described above but using just onsets or offsets revealed qualitatively equivalent impact of color to that reported in the manuscript: Redcone Delay (Onsets) - Time (F_16, 112_ = 83.8; p < 0.0001), color (F_1, 7_ = 6.5; p = 0.038), interaction (F_16, 112_ = 2.5; p = 0.0032); Redcone Delay (Offsets) - Time (F_16, 112_ = 43.8; p < 0.0001), color (F_1, 7_ = 30.8; p = 0.0009), interaction (F_16, 112_ = 2.0; p = 0.022); Redcone Advance (Onsets) - Time (F_16, 112_ = 186.8; p < 0.0001), color (F_1, 7_ = 7.4; p = 0.03), interaction (F_16, 112_ = 1.3; p = 0.19); Redcone Advance (Offsets) - Time (F_16, 112_ = 26.2; p < 0.0001), color (F_1, 7_ = 0.04; p = 0.85), interaction (F_16, 112_ = 2.2; p = 0.0085).

#### 3. Acute phase re-setting

Phase shifts were measured manually from high magnification actograms by three experienced investigators (blinded to stimulus), by extrapolation of a line of best fit through activity onsets to the day of the pulse. Values reported in the manuscript are the average of those obtained by the three investigators (which were reliably in close correspondence; mean ± SD inter-rater variability = 12.7 ± 9.4 min). Subsequent analysis performed by unpaired t test. Data were excluded from this analysis in a few cases when variability in activity onsets made phase-shifts hard to reliably measure (as assessed by at least two of the investigators); this was the case for 1 individual following ‘blue’ delay, 1 individual following ‘blue’ advance and 3 individuals following ‘yellow’ delays (no individuals excluded following ‘yellow’ advances).

#### 4/5. Color-only and Color with low amplitude diurnal lighting changes

In all cases, circadian period was determined by χ^2^-periodogram (as above). For statistical analyses period estimates obtained under LD conditions and following the subsequent experimental manipulations were compared against each other by paired t tests and against an expected period of 24h by one-sample t tests. No data was excluded.

#### 6. Natural Entrainment paradigm

Analyses of diurnal activity patterns were based on the last 48h of each 3 day block (to reduce effects associated with the transition from preceding stimuli), for a total of 10 days of matched ‘natural’ and ‘intensity-only’ cycles for each individual. Analyses of circadian activity patterns were based on the 24h epochs of constant routine immediately following ‘natural’ and ‘intensity-only’ stimulus blocks, providing a total of 5 days for each condition per individual. To determine mean activity waveforms, PIR reported activity profiles for each individual (10 min bins) were first smoothed by a 2h running mean. We then averaged across the relevant days (as indicated above) and normalized by subtracting the daily mean and dividing by the maxima of the resulting time series. Onsets and offsets were determined from daily mean crossings of the 2h smoothed time-series, all other analyses were performed on unsmoothed data and analyzed by paired-test between ‘natural’ and ‘intensity-only’ conditions (or subsequent constant routine as appropriate). Interdaily Stability (a measure of rhythms robustness) was assessed by quantifying the fraction of variance that was accounted for by a stable 24h rhythm [33] using the formula (with *n* representing the total number of data points, *P* the number of time bins):

$$\text{IS}=\frac{n\sum_{h=1}^{P}{\left({\bar{X}}_{h}-\bar{X}\right)}^{2}}{p\sum_{i=1}^{n}{\left(X_{i}-\bar{X}\right)}^{2}}$$

Rhythm amplitude was determined by calculating the percentage of daily activity that occurred outside of the 8h night (or projected night) epoch across the relevant selection of days. Intradaily Variability (a measure of the fragmentation of activity patterns [33],) was assessed using the formula:

$$\text{IV}=\frac{n\sum_{h=2}^{n}{\left(X_{i}-X_{i-1}\right)}^{2}}{\left(n-1\right)\sum_{i=1}^{n}{\left(X_{i}-\bar{X}\right)}^{2}}$$

The day-day similarity between activity patterns was assessed by calculating the mean correlation coefficient (Pearson’s r) between every possible pair of days under the relevant experimental conditions (for diurnal cycles 45 pairs, for constant conditions 10 pairs).

### Data and Code Availability

Raw data and analysis code will be provided upon request by the Lead Contact, Tim Brown (timothy.brown@manchester.ac.uk).

## Supplemental Information

Document S1. Figures S1–S4

Document S2. Article plus Supplemental Information
