# Supplementary material for: Cones Support Alignment to an Inconsistent World by Suppressing Mouse Circadian Responses to the Blue Colors Associated with Twilight
Source: Curr Biol. 2019 Dec 16;29(24):4260–4267.e4. doi: 10.1016/j.cub.2019.10.028 (PMC6926481; doi:10.1016/j.cub.2019.10.028)
Supplement: Document S1. Figures S1–S4 [file mmc1.pdf]

Current Biology, Volume *29*

Supplemental Information

Cones Support Alignment to an Inconsistent World by Suppressing Mouse Circadian Responses to the Blue Colors Associated with Twilight

Joshua W. Mouland, Franck Martial, Alex Watson, Robert J. Lucas, and Timothy M. Brown

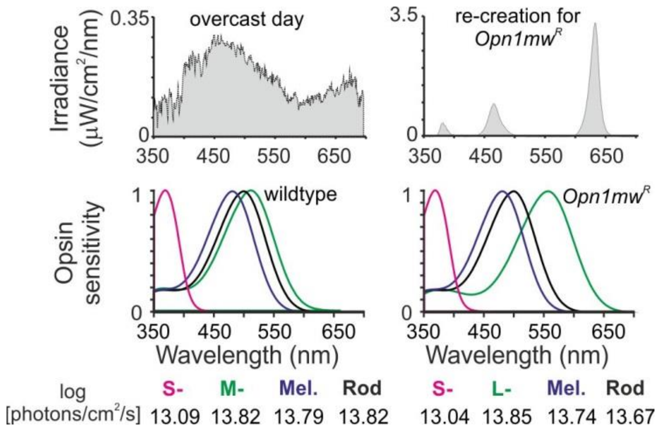

Figure S1. Generation of polychromatic stimuli that re-create a mouse's experience of daylight. Related to Figure 1. Left panels show the spectral distribution of ambient illumination on an overcast day just prior to dusk (from data presented in [S1]) and the corresponding excitation of the four mouse opsin classes. Right panels show the spectral power distribution of a 3-primary polychromatic 'reference' stimulus designed to re-create an equivalent pattern of photoreceptor activation for red cone mice (*Opn1mw*^{R}). Experimental stimuli (illustrated in Figure 1A) were derived from this background by independently modulating the intensity of the three primaries so as to alter the ratio of L- to S-cone opsin activation (and therefore changes to colour so that they appear 'blue' or 'yellow' relative to this reference) without changing excitation of melanopsin, rods or the overall average cone flux.

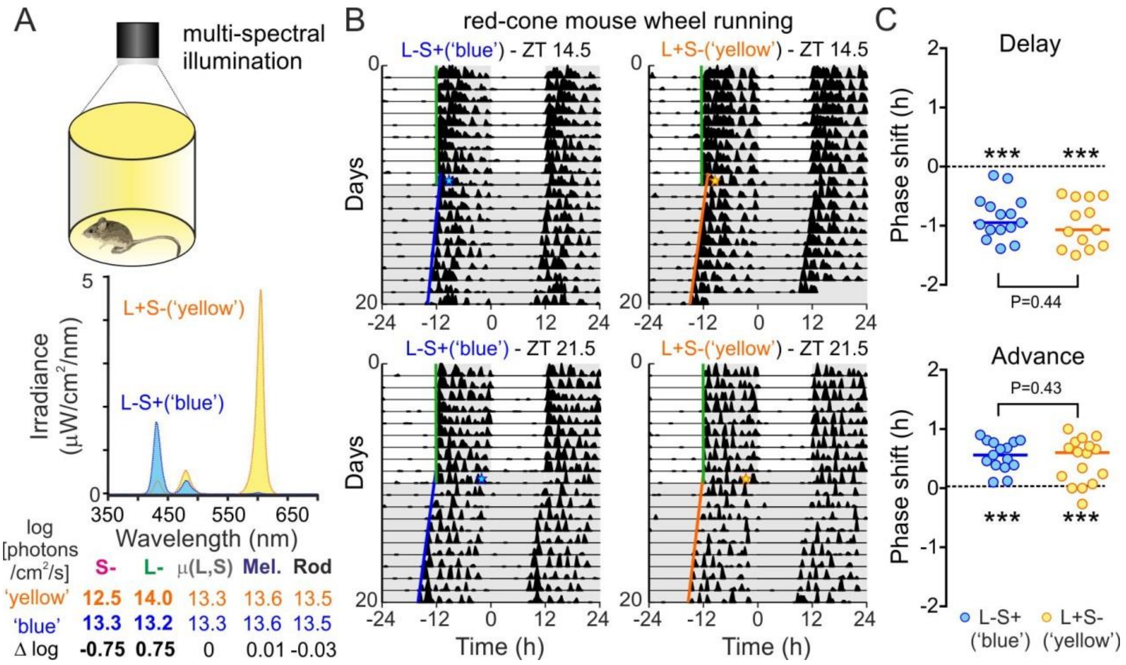

Figure S2. Colour does not overtly modulate acute light-pulse induced clock resetting. Related to Figure 2.

(**A**) Schematic of light exposure apparatus (top) and spectral power distributions and relevant quantification for L-S+('blue') and L+S-('yellow') stimuli.

(**B**) Representative actograms for red-cone mice receiving L-S+('blue') (left) or L+S-('yellow') (right) 5 min light pulses early (top) or late (bottom) in the night, immediately following transfer from LD to constant darkness.

(**C**) Magnitude of phase delays (top) and advances (bottom) for L-S+('blue') and L+S-('yellow') stimuli applied between Zeitgeber time (ZT) 14-15 and 21-22 respectively (n=13-16). Comparison between responses to the two stimuli did not identify significant differences in either case (unpaired t-tests; P=0.44 and P=0.43 respectively). *** represents P<0.001 for one-sample t-tests vs. shift=0h.

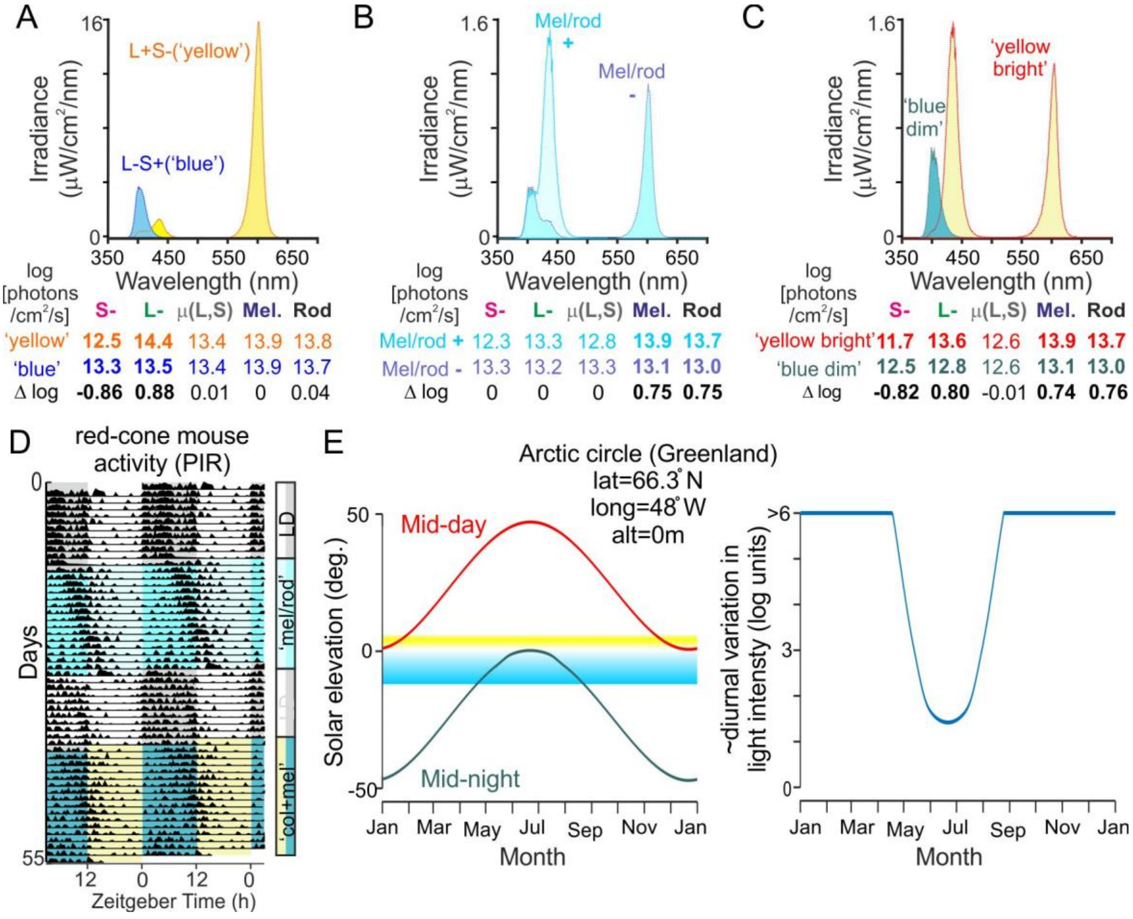

Figure S3. Colour and intensity modulating stimuli and relevance to extreme environments. Related to Figure 3.

(**A-C**) Spectral power distributions and relevant quantification of stimuli designed to vary in 'blue'-'yellow' colour (**A**), melanopsin/rod excitation but not colour (**B**) or both melanopsin/rod excitation and colour (**C**) as used in Figure 3.

(**D**) Actogram for a second red-cone mouse that exhibited partial entrainment under the 'col+mel' conditions.

(**E**) Left panel shows relationship between time of year and solar azimuth at mid-day and mid-night during the arctic summer, right panel shows the corresponding expected diurnal change in light intensity (based on relationship between solar angle and irradiance for rural locations in [S2]). Shaded band in left panel of **E** represents the range of solar elevations where significant colour changes detectable to mammals are expected (from [S1-S3]).

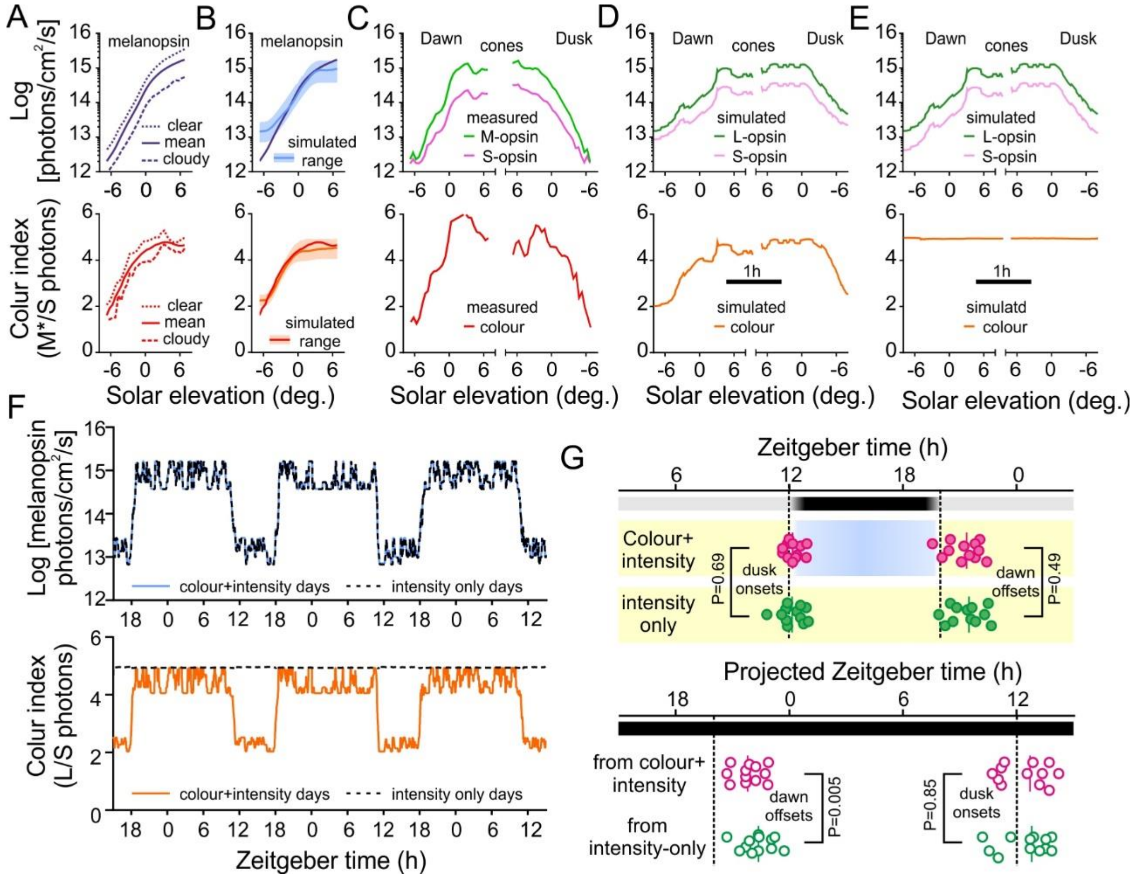

Figure S4. Generation of naturalistic diurnal lighting cycles. Related to Figure 4.

(**A**) Upper panel shows relationship between solar angle and melanopsin excitation under clear and cloudy days (based on Manchester, UK summer; dataset presented in [S1]). Lower panel shows corresponding variations in apparent colour (ratio of M to S-cone opsin excitation).

(**B**) Range of melanopsin excitation and co-occurring colour changes achievable for red-cone mice with our multispectral lighting system, covering much of the natural twilight variation.

(**C**) Representative variation in M and S-cone excitation (and corresponding colour index) for single dawn and dusk transitions measured on days with variable cloud cover (from [S1]).

(**D**) Example of simulated dawn and dusk transitions for red-cone mice incorporating naturalistic cloud-related variations. Scale bar indicates timing over which changes were presented to simulate the extended twilight of a Manchester summer.

(**E**) Simulated dawn dusk transition (matched to those shown in **D**) where colour change is removed to provide a fixed ratio of L- to S-cone opsin excitation.

(**F**) Illustration of a pair of matched 3-day stimulus blocks providing naturalistic changes in colour and intensity or intensity only with continuously varying cloud cover.

(**G**) Timing of activity onsets and offsets (median across all relevant days for each mouse; n=12 mice) under colour+intensity or intensity only days (top) and subsequent 24h epochs of constant conditions (bottom). Dotted lines indicate timing of (projected) dawn and dusk. Data analysed by paired t-tests.

Supplemental references

S1. Walmsley, L., Hanna, L., Mouland, J., Martial, F., West, A., Smedley, A.R., Bechtold, D.A., Webb, A.R., Lucas, R.J., and Brown, T.M. (2015). Colour as a signal for entraining the mammalian circadian clock. PLoS biology *13*, e1002127.

S2. Spitschan, M., Aguirre, G.K., Brainard, D.H., and Sweeney, A.M. (2016). Variation of outdoor illumination as a function of solar elevation and light pollution. Sci Rep *6*, 26756.

S3. Woelders, T., Wams, E.J., Gordijn, M.C.M., Beersma, D.G.M., and Hut, R.A. (2018). Integration of color and intensity increases time signal stability for the human circadian system when sunlight is obscured by clouds. Sci Rep *8*, 15214.
